# Reduction in Ocular Surface Culture Positivity Following Short-Term Treatment with Liposomal Ozonated Oil Eyedrops

**DOI:** 10.3390/clinpract16030059

**Published:** 2026-03-10

**Authors:** Andreea-Talida Tirziu, Maria-Alexandra Preda, Aimee Rodica Chis, Ionela-Iasmina Yasar, Norberth-Istvan Varga, Florin George Horhat, Mihnea Munteanu, Rosca Cosmin

**Affiliations:** 1 Doctoral School, “Victor Babes” University of Medicine and Pharmacy, Eftimie Murgu Square, No. 2, 300041 Timisoara, Romania; andreea.tirziu@umft.ro (A.-T.T.); norberth.varga@umft.ro (N.-I.V.); 2 Ophthalmology Department, “Victor Babes” University of Medicine and Pharmacy, Eftimie Murgu Square, No. 2, 300041 Timisoara, Romania; ionela.yasar@umft.ro (I.-I.Y.); mihnea.munteanu@umft.ro (M.M.); 3 Department of Biochemistry, “Victor Babes” University of Medicine and Pharmacy, Eftimie Murgu Square, No. 2, 300041 Timisoara, Romania; chis.aimee@umft.ro; 4 Multidisciplinary Research Center on Antimicrobial Resistance (MULTI-REZ), “Victor Babes” University of Medicine and Pharmacy, Eftimie Murgu Square, No. 2, 300041 Timisoara, Romania; horhat.florin@umft.ro; 5 Department of Microbiology, “Victor Babes” University of Medicine and Pharmacy, Eftimie Murgu Square, No. 2, 300041 Timisoara, Romania; 6 “Oculens” Ophtalmology Clinic, Calea Turzii, No. 134, 400347 Cluj-Napoca, Romania

**Keywords:** eyedrops, antiseptics, liposomal osonated oil, ocular surface, conjunctival flora, conjunctivitis, keratitis, blepharitis

## Abstract

**Background/Objectives**: The ocular surface is continuously exposed to microorganisms, and disruption of host–microbial balance may lead to infection or postoperative complications. Increasing antimicrobial resistance and biofilm formation have highlighted the need for alternative or complementary non-antibiotic strategies to control ocular surface microbial burden. Liposomal ozonated oil eyedrops have demonstrated antimicrobial and antibiofilm activity in preclinical and preliminary clinical studies. The aim of this study was to evaluate changes in ocular surface microbiological culture results before and after treatment with liposomal ozonated oil eyedrops in a real-world clinical setting. **Methods**: This was a prospective, observational, real-world pre–post study including 101 eyes from 101 patients undergoing ocular surface microbiological sampling in routine clinical practice. Two samples were obtained per patient: Sample I immediately before treatment and Sample II at the routine follow-up visit after short-course treatment with liposomal ozonated oil eyedrops (1 drop, four times daily, for 4 days). The interval between samples ranged from 3 to 5 days (median 3 days). Microbiological cultures were classified as positive or showing no growth. Paired changes in culture positivity were analyzed using McNemar’s exact test. **Results**: At baseline, 87 of 101 samples (86.1%) yielded positive cultures, while 14 (13.9%) showed no growth. Following treatment, culture positivity decreased to 11 of 101 samples (10.9%), with 90 samples (89.1%) showing no growth. Among baseline-positive samples, microbiological clearance was observed in 76 cases (87.4%). No cases converted from culture-negative to culture-positive at follow-up. The reduction in culture positivity after treatment was statistically significant (McNemar’s exact test, *p* < 0.001). Recent antibiotic exposure within 14 days prior to baseline sampling was reported in 8 patients (7.9%). Persistent positive cultures were observed in a minority of cases and were mainly associated with common ocular surface pathogens. **Conclusions**: In routine clinical practice, short-term treatment with liposomal ozonated oil eyedrops was associated with a significant reduction in ocular surface culture positivity over a short follow-up interval.

## 1. Introduction

The ocular surface is continuously exposed to microorganisms derived from the environment and from the patient’s own periocular skin and mucosal flora [1,2]. In healthy individuals, a stable balance between host defenses and resident microorganisms is maintained; however, disruption of this equilibrium may lead to ocular surface colonization, infection, or postoperative complications [3]. Ocular surface infections and microbial colonization play a clinically relevant role in common conditions such as conjunctivitis, blepharitis, keratitis, contact lens–associated complications, and perioperative settings, including cataract surgery and intravitreal procedures [4,5]. Numerous studies have shown that the normal conjunctival flora is dominated by coagulase-negative staphylococci, Staphylococcus aureus, and, to a lesser extent, Gram-negative organisms, which may act as endogenous sources of infection under favorable conditions [6,7,8].

Current strategies for controlling ocular surface microorganisms rely primarily on topical antibiotics and antiseptic agents such as povidone-iodine or chlorhexidine [8,9]. Although these approaches are effective in many settings, they are associated with important limitations. The increasing prevalence of antimicrobial resistance has reduced the efficacy of commonly used topical antibiotics, while repeated or prolonged exposure may further contribute to resistance selection [10,11]. In addition, several ocular pathogens are capable of forming biofilms on the ocular surface and on medical devices such as contact lenses, which markedly reduces susceptibility to antimicrobial agents and promotes persistence and recurrence of infection [12,13,14]. Antiseptic agents, while effective, may be associated with epithelial toxicity and reduced tolerability, limiting their use outside short perioperative applications [12].

Ozonated oils have emerged as a potential antimicrobial approach based on a distinct mechanism of action [4,15,16,17]. Ozonated oils are lipid substrates in which ozone reacts with unsaturated fatty acids to generate stable ozonides that release reactive oxygen species in a controlled manner [4]. This oxidative mechanism induces damage to microbial cell membranes and intracellular components, resulting in broad-spectrum antimicrobial activity against bacteria, fungi, and viruses. Because this action is non-specific and multi-target, the likelihood of inducing antimicrobial resistance is considered low [4,18]. Formulation plays a critical role in ocular applications; incorporation of ozonated oil into liposomal delivery systems improves stability, enhances interaction with microbial membranes, and increases ocular tolerability, making topical ophthalmic use feasible.

Studies have demonstrated that liposomal ozonated oil formulations exhibit significant antimicrobial and antibiofilm activity, reduce bacterial adhesion to human corneal epithelial cells, and may favorably influence bacterial susceptibility to antibiotics [19,20,21]. Clinical investigations have provided preliminary evidence of efficacy and safety, including a significant reduction in conjunctival microbial load in perioperative settings and favorable tolerability profiles [22]. However, most available clinical data are derived from perioperative studies, case series, or adjunctive use, and real-world evidence evaluating microbiological outcomes in routine clinical practice remains limited.

There is a paucity of data in the literature specifically assessing paired pre- and post-treatment microbiological culture outcomes following topical liposomal ozonated oil therapy in a real-world clinical setting. From a clinical standpoint, liposomal ozonated oil eye drops are a general adjunct antiseptic strategy (e.g., perioperative prophylaxis and selected ocular surface conditions) rather than as a replacement for topical antibiotics in sight-threatening microbial keratitis. Accordingly, the present study was designed to evaluate short-term microbiological culture conversion as a surrogate marker of culturable bacterial burden under routine laboratory conditions, not to assess clinical efficacy as a standalone alternative to antibiotics in severe keratitis. At the time of manuscript preparation, registered interventional studies evaluating ozone-based/liposomal ozonated oil eye drops were also listed on ClinicalTrials.gov (e.g., NCT04087733) [23], highlighting ongoing clinical evaluation of this approach. Therefore, the main objective of the present study was to evaluate changes in ocular surface microbiological culture results before and after treatment with liposomal ozonated oil eyedrops in a clinical practice setting. Secondary objectives included describing the distribution of baseline microbial isolates and characterizing organisms identified in persistent positive cultures following treatment.

## 2. Materials and Methods

### 2.1. Study Design and Setting

This study was a prospective, observational, single-center, real-world pre–post study evaluating the change in ocular surface culture results before and after treatment with liposomal ozonated oil eyedrops in routine clinical practice. Patients were enrolled consecutively as part of routine care; there was no randomization and no study-mandated comparator group. Each patient contributed one study eye. Patients were evaluated at the Ophthalmology Clinic of the Emergency Clinical Municipal Hospital of Timisoara, Romania. Data were collected between November 2023 and April 2024. The study population reflects the local catchment area, including both urban and rural residents.

### 2.2. Participants

Eligible patients were adult patients (≥18 years) who underwent ocular surface microbiological sampling and received liposomal ozonated oil eye drops between two predefined sampling time points. Exclusion criteria were: ocular surgery or invasive procedures within the previous three months, active severe ocular infection or trauma, known hypersensitivity to the study formulation, and conditions associated with significant systemic immunosuppression, such as ongoing chemotherapy or uncontrolled HIV infection.

Only one eye per patient was included to avoid within-patient correlation. If both eyes met the eligibility criteria, the first sampled eye was selected.

### 2.3. Intervention

A commercially available liposomal ozonated oil eye drop formulation (Desodrop) was administered as 1 drop, four times daily, for a 4-day treatment period. The timing of follow-up microbiological sampling was determined by routine clinical scheduling and could occur before completion of the 4-day course. No additional study-mandated treatments were introduced during the intervention. Any concomitant ocular or systemic therapies were documented throughout the study period.

This liposomal ozonated oil ophthalmic solution is based on LipozonEye (ozonated sunflower oil and soy phospholipids) and hypromellose (hydroxypropyl methylcellulose); excipients include boric acid, sodium tetraborate, disodium edetate, polyhexamethylene biguanide (PHMB), and purified water. It was selected because it is routinely used in our clinical setting as an antibiotic-sparing supportive antiseptic approach.

At baseline and consequent visits, tolerability was assessed: patients were asked about ocular discomfort (including burning/stinging, foreign-body sensation, and photophobia). Slit-lamp examination was performed, and conjunctival hyperemia and corneal integrity were evaluated; fluorescein staining was used when clinically indicated. Tear film break-up time (TBUT) and Schirmer testing were performed when clinically required. Discontinuation and adherence to the prescribed QID regimen were assessed at follow-up based on patient report and clinical documentation.

### 2.4. Microbiological Sampling and Culture Procedures

Two samples were obtained per patient: the first sample was collected immediately before initiating liposomal ozonated oil eye drops, and the second sample (follow-up) was collected shortly after. In routine clinical practice, the follow-up sampling was typically scheduled approximately 3–4 days after baseline (range 3–5 days), but patients continued the eyedrops to complete the full 4-day regimen irrespective of the sampling day.

Sampling was performed from the inferior conjunctival fornix using a sterile swab. Sampling was performed prior to any ocular surface manipulation or instillation of other topical agents at the visit (e.g., irrigation, topical anesthetic, fluorescein or other diagnostic drops), when applicable.

Swabs were transported dry (without transport medium) and delivered to the microbiology laboratory within 30 min. In the laboratory, swabs were streaked onto blood agar and chocolate agar plates and onto a chromogenic agar medium. Plates were incubated at 36–37 °C under aerobic conditions (with CO_2_ enrichment for chocolate agar, according to laboratory protocol). Plates were inspected at approximately 24 h. Plates with no visible colonies at 24 h were re-inspected at approximately 48 h before being finalized as “no growth”.

Culture results were recorded qualitatively for aerobic and fastidious bacteria. Anaerobic culture conditions, fungal media (e.g., Sabouraud), parasitological cultures (e.g., Acanthamoeba), and viral testing were not performed as part of the routine conjunctival culture panel in this study.

Bacterial identification was performed according to routine laboratory protocols using commercially available biochemical and automated systems, including API 10 S and API NH strips and the VITEK 2 automated identification platform (bioMérieux, Marcy-l’Étoile, France), as well as latex agglutination assays for Staphylococcus aureus (Staphytect Plus; Oxoid Ltd., Basingstoke, UK) and streptococcal Lancefield grouping (Streptococcal Grouping Kit/Streptex; Oxoid Ltd., Basingstoke, UK). Identification outputs were interpreted in accordance with manufacturer instructions; when low-discrimination profiles occurred, additional confirmatory testing was performed as per routine laboratory practice.

A “positive culture” was defined as any bacterial growth on any of the inoculated media at the final plate inspection. “No growth” indicated the absence of visible colonies on all media at the final inspection. Colony counts or semiquantitative grading were not performed, and no enrichment broth was used.

### 2.5. Statistical Analysis

Categorical variables were summarized as counts and percentages. Continuous variables were summarized as mean ± SD or median (range), as appropriate. The change in culture positivity between samples was analyzed using McNemar’s exact test for paired binary outcomes. The clearance proportion among baseline-positive samples was reported with 95% confidence intervals. Analyses were performed using SPSS version 27 (IBM, Armonk, NY, USA). Statistical significance was defined as *p* < 0.05 (two-sided).

No a priori sample size or power calculation was performed, as this was an exploratory real-world pre–post study. The sample size reflects the number of consecutive eligible patients evaluated during the predefined study period. To facilitate interpretation of the primary endpoint, a post hoc (achieved) power analysis was performed for McNemar’s exact test (two-sided α = 0.05) using the observed discordant pair proportions from the paired culture table (positive → negative and negative → positive).

### 2.6. Ethical Considerations

The study was approved by the Ethics Committee of the Clinical Municipal Hospital, Timisoara (approval number E-6876 from 27 November 2023) and the Doctoral School of the “Victor Babes” University of Medicine and Pharmacy (number 89/01.10.2023_rev2025). Written informed consent was obtained from every patient. All data were de-identified prior to analysis.

This manuscript was originally written by the authors in English, and ChatGPT (version 5.1, OpenAI, San Francisco, CA, USA) was used for the sole purpose of improving the language. The authors take full responsibility for the content of this manuscript.

## 3. Results

### 3.1. Study Population

A total of 101 eyes from 101 patients were included in the analysis. The mean age of the study population was 67.3 ± 10.9 years (median 68.5 years; range 38–89), with 52 males (51.5%) and 49 females (48.5%). Fifty-four eyes (53.5%) were right eyes and 47 (46.5%) were left eyes. Sixty-three patients (62.4%) resided in urban areas and 38 (37.6%) in rural areas. Eight patients (7.9%) reported topical and/or systemic antibiotic use within the 14 days preceding baseline sampling, while 93 patients (92.1%) had not received antibiotics during that period. The interval between baseline sampling (pre-treatment) and follow-up sampling ranged from 3 to 5 days. Table 1 presents baseline characteristics of the study population.

### 3.2. Baseline Microbiological Findings

At baseline, 87 of 101 samples (86.1%) yielded positive bacterial cultures, while 14 samples (13.9%) showed no growth. The most frequently isolated organisms at baseline were *coagulase-negative staphylococci*, predominantly *Staphylococcus haemolyticus*, followed by *Enterococcus faecalis* and *Escherichia coli*. Other isolated species included *Staphylococcus aureus* (including methicillin-resistant strains), *Pseudomonas aeruginosa*, *Moraxella catarrhalis*, and other *Gram-negative bacilli* at lower frequencies. Baseline microbiological isolates and their distribution are summarized in Table 2.

### 3.3. Microbiological Clearance After Treatment

Following treatment with liposomal ozonated oil eyedrops, a marked reduction in culture positivity was observed. At follow-up, 90 of 101 samples (89.1%) showed no bacterial growth, while 11 samples (10.9%) remained culture-positive.

Paired analysis demonstrated:

- 14 eyes that were culture-negative at baseline remained negative at follow-up.
- 76 eyes that were culture-positive at baseline became culture-negative after treatment.
- 11 eyes remained culture-positive at follow-up.
- No cases of culture conversion from negative to positive were observed.

Among eyes with a positive baseline culture, culture conversion from growth to no growth occurred in 76/87 cases (87.4%). Given the qualitative nature of the cultures, this “clearance” reflects conversion to no detectable growth under the culture conditions described above. McNemar’s exact test confirmed a statistically significant reduction in culture positivity after treatment (*p* < 0.001). Based on the observed discordant pairs (positive → negative = 76; negative → positive = 0; n = 101), the post hoc achieved power for the primary McNemar comparison at α = 0.05 (two-sided), assuming the true discordant-pair direction and magnitude equal to that observed, was approximately 1.00 (>99.99%). We note that achieved post hoc power is intrinsically related to the observed *p*-value; therefore, we also report the discordant counts explicitly as the primary effect descriptor. Paired microbiological outcomes are presented in Table 3.

Regarding tolerability during treatment, no burning was reported in patients receiving liposomal ozonated oil eyedrops alone. No foreign-body sensation or photophobia was reported during the treatment period. No clinically significant conjunctival hyperemia was noted at follow-up. Fluorescein staining performed after drop administration did not reveal corneal epithelial defects attributable to treatment, supporting preservation of epithelial integrity. When clinically assessed, TBUT did not show clinically meaningful deterioration compared with baseline. When Schirmer testing was performed as part of routine care, no clinically significant reduction in tear production attributable to treatment was observed. No patients discontinued treatment due to intolerance, and adherence to the prescribed QID regimen was satisfactory during the study period.

### 3.4. Persistent Positive Cultures and Organism Distribution

Among the 11 eyes with persistent positive cultures, the most frequently identified organism at follow-up was *Staphylococcus aureus*, followed by *Escherichia coli* and *Moraxella catarrhalis*. In a small number of cases, the organism identified at follow-up differed from the baseline isolate, likely reflecting either strain-level variation or differences in laboratory reporting categories.

No emergence of previously undetected organisms was observed in eyes that were culture-negative at baseline.

Persistent positive isolates are detailed in Table 4.

### 3.5. Exploratory Observations

Among patients who had not received antibiotics within 14 days prior to baseline sampling, 10 of 93 (10.8%) remained culture-positive at follow-up, compared with 1 of 8 (12.5%) among those reporting recent antibiotic exposure. Given the small number of patients with prior antibiotic use, no inferential statistical analysis was performed for this comparison.

## 4. Discussion

In this real-world pre-post study of 101 eyes, treatment with liposomal ozonated oil eyedrops was associated with a marked reduction in culture positivity. Baseline cultures were positive in 86.1% of cases, whereas only 10.9% remained positive after treatment, corresponding to an 87.4% clearance rate among baseline-positive samples. Notably, no cases converted from culture-negative to culture-positive at follow-up, supporting an overall shift toward “no growth” results over a short treatment interval.

Interpretation of rapid culture conversion on the ocular surface requires consideration of ocular surface biology. The conjunctiva is a low-biomass ecosystem, and a shift from “growth” to “no growth” over days may reflect suppression of culturable organisms below the detection threshold of standard culture conditions rather than complete eradication. In addition, resident commensals contribute to immune education and colonization resistance; therefore, reduced growth on culture is not synonymous with restoration of a healthy ocular surface microbiome and could have uncertain longer-term consequences if used indiscriminately. In this context, the short-course regimen evaluated here may be advantageous by aiming to transiently reduce microbial burden while limiting prolonged exposure to an oxidative antiseptic; however, durability and potential dysbiosis risk require longer follow-up with clinical endpoints and microbiome-focused methods (quantitative/semiquantitative culture and sequencing-based approaches) [1,2,3,7].

The magnitude and direction of this microbiological clearance are consistent with the published biological activity of liposomal ozonated oil formulations. Preclinical evidence indicates that these formulations have broad antimicrobial effects and can reduce biofilm formation and eradicate established biofilms, while also decreasing bacterial adhesion to human corneal epithelial cells and potentially improving susceptibility to antibiotics—mechanisms that may plausibly reduce microbial persistence on the ocular surface [19,20]. In particular, Gentili et al. reported reductions in biofilm formation, a decrease in bacterial adhesion to corneal cells, and effects on antibiotic susceptibility patterns in vitro, supporting an anti-persistence profile beyond simple planktonic killing [19]. Antibiofilm activity on relevant substrates, including contact lens–associated models, has also been demonstrated, alongside cellular responses consistent with repair-related activity [20]. Additional in vitro data have shown antimicrobial activity of ozonated oil liposomal eyedrops against multidrug-resistant bacteria and effects on ocular epithelial cells and potential ocular pathogens, further supporting plausibility across a clinically relevant spectrum [18,21].

Our findings also align with prior clinical evidence in perioperative settings. In the ELOOM phase 4 paired-eye cataract surgery study, topical liposomal ozonated oil administered for three days significantly reduced conjunctival microbial burden and was associated with a high proportion of “zero-growth” outcomes and excellent tolerability [22]. While that study quantified colony-forming units and used contralateral-eye controls, whereas our outcome was binary culture conversion in routine practice, both lines of evidence point toward a meaningful reduction in ocular microbial load after short-course treatment [22]. Case-based clinical reports and small case series in anterior segment conditions similarly describe clinical feasibility and supportive outcomes when liposomal ozonated oil is used as adjunctive therapy, although these reports are inherently limited by non-controlled design [16,17]. A similar paired-sample microbiological approach has been applied to other non-antibiotic antiseptic strategies, such as hypochlorous acid–based eyelid hygiene solutions, which demonstrated a >99% reduction in staphylococcal load within minutes while preserving overall microbial diversity, supporting the clinical relevance of culture-based pre–post assessments in real-world settings [24]. Taken together, our results extend prior work by providing real-world paired microbiological outcomes showing high rates of culture conversion to no growth in routine clinical practice.

Safety and tolerability are clinically important considerations when introducing non-antibiotic antiseptic strategies on the ocular surface. Based on the oxidative mechanism of ozonated oils and general experience with topical ocular antiseptics, anticipated local adverse effects include transient stinging/burning, tearing, conjunctival hyperemia, foreign-body sensation, and, less commonly, ocular surface irritation or hypersensitivity reactions. In the present real-world setting, patients with known hypersensitivity to the formulation were excluded, and follow-up occurred within a short interval during which ocular surface status and tolerability were assessed as part of routine clinical evaluation. In our cohort, no clinically significant adverse effects were observed. This aligns with previous clinical literature on liposomal ozonated oil formulations, that similarly describes favorable tolerability with short-course use [16,17,22].

Persistent culture positivity was observed in a minority of cases, with follow-up isolates enriched for organisms commonly implicated in ocular surface disease, including *Staphylococcus aureus* and Gram-negative bacteria. A few non-mutually exclusive explanations are possible. First, organism- and host-related factors (e.g., baseline burden, local microenvironment, underlying ocular surface disease, or concomitant therapies) may influence whether organisms remain detectable after a short treatment window. Second, biofilm-associated colonization and device-related biofilms are recognized contributors to persistence and recurrence and may reduce apparent culture conversion [14]. Third, antibiotic exposure is known to alter ocular flora and may contribute to selection pressure and persistence of harder-to-eradicate organisms; however, in our cohort, recent antibiotic exposure (within 14 days prior to baseline sampling) was rare (7.9%), and the small exposed subgroup limits inference regarding its impact on baseline microbiology or persistence patterns. Although our study did not include susceptibility testing or direct biofilm measurements, prior mechanistic data suggest that liposomal ozonated oil may reduce biofilm formation and limit resistance selection pressure, which could be advantageous in this setting [19]. Importantly, persistent culture positivity at short follow-up does not necessarily indicate treatment failure. The ocular surface and adnexa are open ecosystems with continuous microbial seeding from the lid margin and periocular skin, and complete ‘sterilization’ is unlikely even when a large reduction in bacterial load is achieved. In a periocular hygiene study, a single antiseptic application produced a marked reduction in staphylococcal CFU while commensals persisted and different strains were recoverable shortly thereafter, supporting rapid recolonization after superficial decontamination [24].

This study has limitations that should be considered. The design was an observational pre–post study without an untreated/placebo/standard-of-care control group; therefore, causal inference is limited and the observed reduction could, in principle, be influenced by spontaneous fluctuation, regression to the mean, or delayed effects of prior therapies. In addition, no a priori sample size calculation was undertaken; therefore, the study should be interpreted as hypothesis-generating. Outcomes were binary (growth vs. no growth) rather than quantitative colony counts, preventing estimation of the magnitude of bacterial load reduction and limiting comparability with CFU-based studies. Although our dataset contains data about comorbidities and concomitant treatments, we chose not to include these variables in the primary analysis. Microbiological susceptibility testing and biofilm assessment were not performed, limiting inference about organism-specific responses and resistance-related questions. The culture approach reflected routine aerobic culture conditions and is not comprehensive for the ocular surface ecosystem; slow-growing organisms, anaerobes, fungi, and non-bacterial pathogens were not specifically targeted, and no enrichment broth, extended incubation, anaerobic conditions, or molecular microbiome profiling were performed. From a broader ocular surface microbiome perspective, complete eradication of resident microorganisms may neither be achievable nor desirable, as contemporary evidence emphasizes the importance of preserving microbial balance while reducing pathogenic overgrowth, rather than pursuing indiscriminate sterilization.

## 5. Conclusions

In this real-world paired pre–post study, short-term treatment with liposomal ozonated oil eyedrops was associated with a marked reduction in the proportion of conjunctival cultures showing bacterial growth. A high proportion of baseline-positive cultures converted to “no growth” at follow-up, with no observed conversion from “no growth” to “growth” over the short sampling interval. Because the study lacked a control group and relied on qualitative culture outcomes, these findings should be interpreted as evidence of short-term culture conversion under routine laboratory conditions rather than proof of eradication or restoration of a healthy ocular surface microbiome. These microbiological findings should not be interpreted as evidence to replace topical antibiotics, but rather as real-world evidence supporting further controlled evaluation of liposomal ozonated oil as an adjunctive, antibiotic-sparing strategy for reducing ocular surface microbial burden.

## Figures and Tables

**Table 1:** Main Characteristics of the Study Population.

Characteristic	Value
Number of eyes/patients	101
Age, mean ± SD (years)	67.3 ± 10.9
Age, median (range)	68.5 (38–89)
Sex, n (%)	
Male	52 (51.5)
Female	49 (48.5)
Eye, n (%)	
Right	54 (53.5)
Left	47 (46.5)
Provenience, n (%)	
Urban	63 (62.4)
Rural	38 (37.6)
Antibiotic use within 14 days before baseline, n (%)	
No	93 (92.1)
Yes	8 (7.9)
Interval between Sample I and Sample II, days	
Median (range)	3 (3–5)
3 days	73
4 days	15
5 days	13

**Table 2:** Baseline Microbiological Isolates.

Microorganism	n (%)
*Staphylococcus haemolyticus*	27 (26.7)
*Enterococcus faecalis*	18 (17.8)
*Escherichia coli*	15 (14.9)
*Staphylococcus aureus* (including MRSA)	9 (8.9)
*Pseudomonas aeruginosa*	6 (5.9)
*Moraxella catarrhalis*	5 (5.0)
Other Gram-negative bacilli	7 (6.9)
Mixed or unspecified bacteria	10 (9.9)
No growth	14 (13.9)
Total	101 (100)

**Table 3:** Paired Microbiological Outcomes Before and After Treatment.

Sample I → Sample II	n
Negative → Negative	14
Negative → Positive	0
Positive → Negative (cleared)	76
Positive → Positive (persistent)	11
Total	101

**Table 4:** Persistent Positive Cultures at Follow-up.

Microorganism	n
*Staphylococcus aureus*	5
*Escherichia coli*	3
*Moraxella catarrhalis*	2
Gram-negative bacillus (unspecified)	1
Total	11

## Data Availability

The data presented in this study are available on request from the corresponding author. The data are not publicly available due to legal and ethical considerations.
